# CDK1 and HSP90AA1 Appear as the Novel Regulatory Genes in Non-Small Cell Lung Cancer: A Bioinformatics Approach

**DOI:** 10.3390/jpm12030393

**Published:** 2022-03-04

**Authors:** Nirjhar Bhattacharyya, Samriddhi Gupta, Shubham Sharma, Aman Soni, Sali Abubaker Bagabir, Malini Bhattacharyya, Atreyee Mukherjee, Atiah H. Almalki, Mustfa F. Alkhanani, Shafiul Haque, Ashwini Kumar Ray, Md. Zubbair Malik

**Affiliations:** 1 School of Biotechnology, Jawaharlal Nehru University, New Delhi 110067, India; nirjhar.suvo.97@gmail.com; 2 Department of Biochemistry, University of Hyderabad, Hyderabad 500046, India; samriddhi1512@gmail.com; 3 School of Computational and Integrative Sciences, Jawaharlal Nehru University, New Delhi 110067, India; shubha65_sit@jnu.ac.in (S.S.); aman42_sit@jnu.ac.in (A.S.); 4 Department of Medical Laboratory Technology, Faculty of Applied Medical Sciences, Jazan University, Jazan 45142, Saudi Arabia; sbagabir@jazanu.edu.sa; 5 Department of Environmental Plant Biology, Hemvati Nandan Bahuguna, Garhwal Central University, Srinagar 246174, India; malinipresi2019@gmail.com; 6 Department of Life Sciences, Presidency University, Kolkata 700073, India; atreyee170597@gmail.com; 7 Department of Pharmaceutical Chemistry, College of Pharmacy, Taif University, Taif 21944, Saudi Arabia; ahalmalki@tu.edu.sa; 8 Addiction and Neuroscience Research Unit, College of Pharmacy, Taif University, Taif 21944, Saudi Arabia; 9 Emergency Service Department, College of Applied Sciences, Al Maarefa University, Riyadh 11597, Saudi Arabia; mkhanani@mcst.edu.sa; 10 Research and Scientific Studies Unit, College of Nursing and Allied Health Sciences, Jazan University, Jazan 45142, Saudi Arabia; shafiul.haque@hotmail.com; 11 Faculty of Medicine, Bursa Uludağ University, Görükle Campus, Bursa 16059, Turkey; 12 Department of Environmental Studies, University Delhi, New Delhi 110007, India

**Keywords:** non-small cell lung cancer, key regulator, differentially expressed genes, protein–protein interaction

## Abstract

Lung cancer is one of the most invasive cancers affecting over a million of the population. Non-small cell lung cancer (NSCLC) constitutes up to 85% of all lung cancer cases, and therefore, it is essential to identify predictive biomarkers of NSCLC for therapeutic purposes. Here we use a network theoretical approach to investigate the complex behavior of the NSCLC gene-regulatory interactions. We have used eight NSCLC microarray datasets GSE19188, GSE118370, GSE10072, GSE101929, GSE7670, GSE33532, GSE31547, and GSE31210 and meta-analyzed them to find differentially expressed genes (DEGs) and further constructed a protein–protein interaction (PPI) network. We analyzed its topological properties and identified significant modules of the PPI network using cytoscape network analyzer and MCODE plug-in. From the PPI network, top ten genes of each of the six topological properties like closeness centrality, maximal clique centrality (MCC), Maximum Neighborhood Component (MNC), radiality, EPC (Edge Percolated Component) and bottleneck were considered for key regulator identification. We further compared them with top ten hub genes (those with the highest degrees) to find key regulator (KR) genes. We found that two genes, CDK1 and HSP90AA1, were common in the analysis suggesting a significant regulatory role of CDK1 and HSP90AA1 in non-small cell lung cancer. Our study using a network theoretical approach, as a summary, suggests CDK1 and HSP90AA1 as key regulator genes in complex NSCLC network.

## 1. Introduction

Lung Cancer is one of the most invasive cancer types causing more than 1.38 million deaths worldwide [1]. Non-Small Cell Lung Cancer (NSCLC) is a kind of epithelial lung cancer. It is markedly different from Small Cell Lung Carcinoma (SCLC), and accounts for about 85% of all lung cancer cases [2]. NSCLCs can be further sub-categorized into adenocarcinoma (32–40%), squamous cell carcinoma (25–30%), and large cell carcinoma (8–16%) based on the type of lung cells undergoing uncontrolled proliferation [3]. In adenocarcinomas, cells that secrete mucus proliferate uncontrollably. This type of cancer occurs mainly among non-smokers [4]. It is more common in women than in men and has a higher probability of occurring in younger people, among other types of lung cancers. It is usually found in the outer parts of the lung; thus, it is likely to be detected before it metastasizes [5].

On the other hand, in squamous cell carcinoma, flat squamous cells that line the inside of the airways in the lungs undergo proliferation. Here, the tumor is usually found in the central part of the lungs, near the bronchi. In large cell carcinoma, the tumor can grow in any part of the lung [6]. It grows and spreads very fast, making it harder to treat. Other subtypes of NSCLCs, such as adeno squamous carcinoma and sarcomatoid carcinoma, are relatively less common.

Out of various reasons, smoking history is highly correlated to the pathogenesis of lung cancer [7]. It is, so far, the most crucial risk factor for lung cancer. Cigarette smoke contains more than 6000 components, most of which result in DNA damage [7,8]. Moreover, other sources of lung cancer include subjection to passive smoke, radon, exposure to materials such as soot, beryllium, nickel, chromium, asbestos, or tar, genetic predisposition to lung cancer, and air pollution [9,10].

DNA damage appears to be the primary reason for pathogenesis in multiple different cancers at the genomic level, let alone NSCLC cases. Even though the DNA repair system can repair most of the damage, frequent exposure to smoke and other causative factors makes it vulnerable [11]. Additionally, epigenetic gene silencing of DNA repair genes can happen during incompletely finished repair sites formed during DNA double-strand breaks or other DNA damage repair [12,13]. The epigenetic gene silencing of DNA repair genes plays a crucial role in molecular pathogenesis of NSCLC. At minimum, nine DNA repair genes that usually function in DNA repair pathways are often suppressed by promoter hypermethylation in NSCLC. They are namely NEIL1, WRN, MGMT, ATM, MLH1, MSH2, BRCA1, BRCA2, and XRCC5 [14,15,16,17,18,19,20]. FEN1, a component of the DNA repair pathway, is expressed at an increased level due to hypomethylation at its promoter region in NSCLC [21]. The complex molecular underpinning of NSCLC is yet to be fully understood for better treatment approaches and drug target identification.

There are not many full-proof treatment options available. Current treatment options include surgical intervention at the early stages like radical mastectomy. Cisplatin-based chemotherapy coupled with radiotherapy has been the go-to treatment with the advancement in the cancer stages and onset of metastasis. However, the multimodal approach is taken in certain cases, where third generation cytotoxic and cytostatic agents such as anti-VGFR and anti-EGFR drugs are prescribed [22].

Thus, further research into recognizing new players, drugs, and combinatorial therapies is necessary to expand the clinical interest to a broader patient population and better outcomes in NSCLC. This study utilizes a network theoretical approach to unveil the complexity of non-small cell lung cancer.

## 2. Materials and Methods

### 2.1. Data Collection

Non-small cell lung cancer data sets were retrieved from Gene Expression Omnibus (GEO), NCBI (GEO, https://www.ncbi.nlm.nih.gov/geo/, accessed on 13 September 2020). A total of eight datasets, GSE19188 (65 control and 91 cancer patients’ samples), GSE118370 (six control and six cancer patients’ samples), GSE10072 (49 control and 58 cancer patients’ samples), GSE101929 (34 control and 32 cancer patients’ samples), GSE7670 (27 control and 27 cancer patients’ samples), GSE33532 (20 control and 80 cancer patients’ samples), GSE31547 (20 control and 30 cancer patients’ samples), GSE31210 (20 control and 226 cancer patients’ samples), were taken into this study. The description of datasets and controls and patient numbers are described in Table 1.

### 2.2. Meta-Analysis of the Datasets

**Figure 1:**
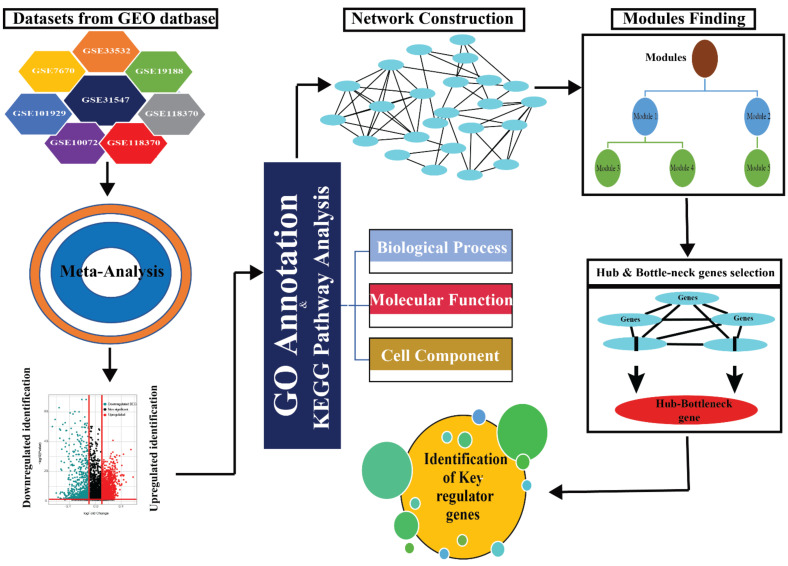
Systematic workflow of the methodology.

The summary of the methodology of the integrated analysis of this study is provided in Figure 1. Meta-analysis of the datasets was conducted using the codes obtained from ImaGEO server R.4.0.2 [32]. The codes received from ImaGEO were further modified using quantile normalization between arrays. The meta-analysis was conducted using the max *p* value method with Benjamini Hochberg F.D.R. value less than 0.05 as significant. The DEGs between cases and control were identified using the R limma package, with a |log2FC| > 0.1 and an adjusted *p*-value < 0.05.

### 2.3. Gene Ontology Analysis

The GO annotation and KEGG pathway enrichment details of the DEGs reported previously were obtained from two different databases, the Database for Annotation Visualization and Integrated Discovery (DAVID; https://david.ncifcrf.gov/) and g: Profiler (https://biit.cs.ut.ee/gprofiler/gost). Statistical significance was attributed to findings with a *p*-value less than 0.05 for ontology terms retrieved from DAVID. For g: Profiler web server Benjamini Hochberg FDR (FDR < 0.05) was used to find significant ontology terms [33]. (The final ontology analysis was carried with g: Profiler (https://biit.cs.ut.ee/gprofiler/gost) and plotted). Both the servers were accessed on 15 September 2020.

### 2.4. PPI Network Construction

Network analysis is critical for providing information about the regulatory roles of the genes by utilizing protein–protein interaction data [34]. The study of the PPI network of DEGs was carried out using STRING database (http://string-db.org/ accessed on 13 September 2020) [35]. The differentially expressed genes (DEGs) were submitted to the STRING database for PPI network construction. Furthermore, the network generated was uploaded to the Cytoscape software for gene network visualization and downstream analysis for key regulator identification using various plug-ins like CytoHubba, CytoNCA, etc.

### 2.5. Network Analysis

The analysis of the topological properties of the network was carried out using the Cytohubba plug-in network analyzer in Cytoscape 3.7. Another plug-in, CytoNCA, was used to calculate the EigenVector values. Modules of the primary network were computed using the “leading eigenvector method” algorithm using the “igraph” package in R.4.0.2 [36]. Additionally, using default parameters (degree cutoff 2, node score cutoff 2, K-core 2, and max depth = 100), the MCODE plug-in of Cytoscape was used to define possible functional modules in the PPI network [37].

### 2.6. Key Regulator Gene Identification

ACytoscape plug-in, Cytohubba was used for identifying key regulator genes (KR) based on several topological properties. The top-ten genes with the highest closeness centrality, Bottleneck, MCC (Maximal Clique Centrality), MNC (Maximum Neighborhood Component), radiality, EPC (Edge Percolated Component) were used to extract key regulator genes. Furthermore, top modules were identified using MCODE, a Cytoscape plug-in (MCODE score ≥ 5). Commons gene for the above mentioned six topological properties were traced to top ten hub genes (genes with maximum degrees) to ensure their reliability as key regulators of the NSCLC gene regulatory network. 

### 2.7. Survival Plot Analysis of the Driver Gene

The GEPIA data tool (http://gepia.cancer-pku.cn/, accessed on 15 September 2020) enables researchers to examine functional genomic datasets for associations between genomic and phenotypic variables. This tool was used to see whether the expression of key genes was linked to the survival of NSCLC patients from the TCGA data. The lung cancer patients were divided into two classes based on median gene expression values. Their overall survivals (OSs) were evaluated using the Kaplan–Meier approach with a log-rank test for obtaining the survival plot of the key regulator or driver gene. We validated the key genes with a box plot, analyzed the pathological stage and transcript per million. The *p* < 0.05 was considered statistically significant. The protein expression pattern of key genes in tumor and normal tissues was validated using the Human Protein Atlas (HPA) database (https://www.proteinatlas.org/, accessed on 8 January 2022).

## 3. Results

### 3.1. Identification of Differentially Expressed Genes and Meta-Analysis

Meta-analysis was performed using the ImaGeo server with eight microarray datasets of non-small cell lung cancer that yielded 4535 genes (Supplementary File S1). This meta-analysis was carried out using Wilkinson’s method or maximum *p*-value method, which is the most restrictive one and is used to identify most robust genes [38]. It considers the maximum *p*-value across the datasets, max (p1, p2, …, pi, pk), which is distributed as beta (k,1) under the null hypothesis [32,39]. After meta-analysis, the fold change of a gene is calculated as the average of fold changes of that gene across all studies. Therefore, the fold change of each gene gets reduced after meta-analysis as compared to its fold changes in individual datasets. We considered an adjusted *p*-value less than 0.05 and an arbitrary threshold of logFC>0.1 and logFC<−0.1 to denote up and downregulated genes. After the analysis between NSCLC (non-small cell lung cancer) and NC (non-cancer) cohorts, a total of 2700 differentially expressed genes were identified, including 1842 genes as upregulated and 858 genes downregulated.

**Figure 2:**
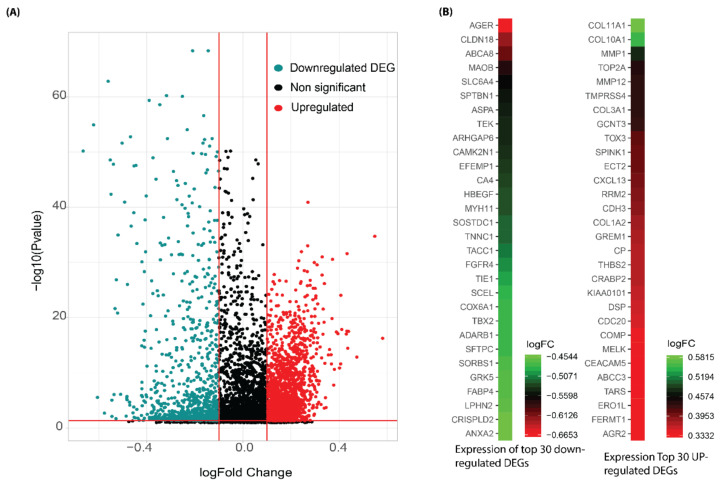
(**A**) Volcano plot distribution highlighting 2700 meta-DEGs between normal and tumor samples. The red and cyan colored points denote the up (1842) and downregulated (858) meta-DEGs. All the black colored points denote non-significant genes. The x and y axes represent the log2FC and −log10 *p*-value, respectively. (**B**) The heatmap denotes the gene expression of top-30 downregulated and upregulated and DEGs based on fold changes. DEGs identification with the volcano plot in a microarray represented the top DEGs in lung cancer cases and controls. The top DEGs follow the logFC value and *p*-value criterion.

The volcano plot shows an overview of differentially expressed genes. (Figure 2A). The top 30 upregulated and downregulated DEGs based on fold changes are shown in Figure 2B.

### 3.2. Gene Ontology Analysis of Differentially Expressed Genes

The g: Profiler server was used for the functional and pathway enrichment analysis of selected DEGs. The ontology terms are categorized as the biological process (BP), cellular component (CC), molecular function (MF) and KEGG pathways. The results were considered statistically significant if Benjamini Hochberg FDR was lesser than 0.05. The top 30 GO terms of the upregulated and downregulated DEGs are compiled in Supplementary File S2 and File S3, respectively.

**Figure 3:**
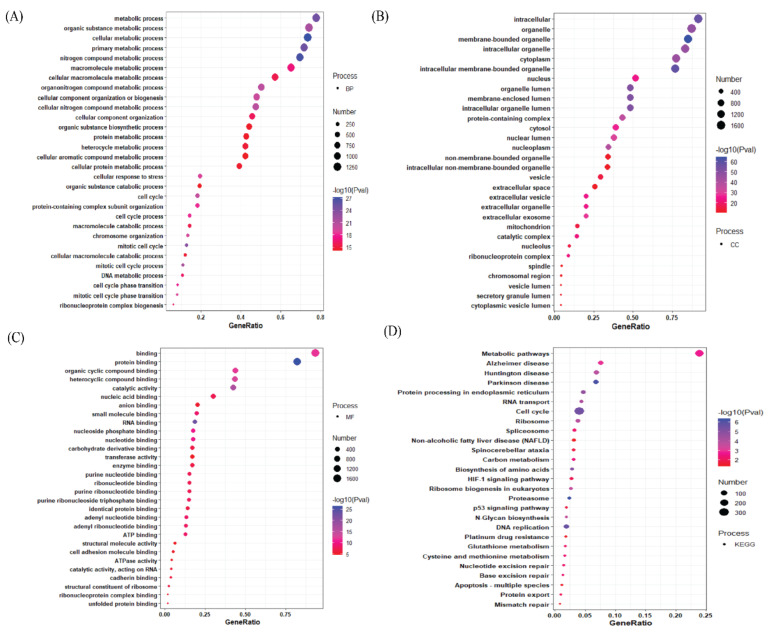
Gene enrichment analyses of upregulated DEGs: (**A**) The upregulated DEGs’ top30 enriched GO terms with logFC > |0.1| for each of the biological processes (BP). (**B**) The upregulated DEGs’ top30 enriched GO cellular component (CC). (**C**) The upregulated DEGs’ top-30 enriched GO molecular function (MF). (**D**) The upregulated DEGs’ top30 enriched GO KEGG pathway (KP). Gene Ratio is defined as the ratio between intersection size and query size. The number refers to interaction size, i.e., the number of genes corresponding to an ontology term and Gene Ratio denotes ratio between intersection and query size.

**Figure 4:**
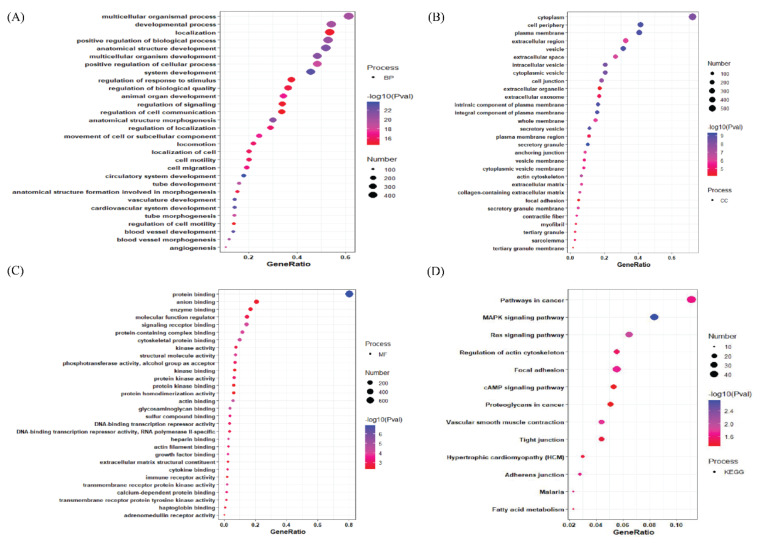
The gene enrichment analysis (**A**) The downregulated DEGs’ top30 enriched GO terms with logFC > |0.1| for each of the biological processes (BP). (**B**) The downregulated DEGs’ top30 enriched GO cellular component (CC). (**C**) The downregulated DEGs’ top 30enriched GO molecular function (MF). (**D**) The downregulated DEGs’ top30 enriched GO KEGG pathway (KP). Gene Ratio is defined as the ratio between intersection size and query size. The number refers to interaction size, i.e., the number of genes corresponding to an ontology term.

The bubble plot demonstrates that the top most upregulated genes are mainly involved in the metabolic process, organic substance metabolic process, and cellular metabolic process (Figure 3A). Furthermore, the top-most upregulated genes are present in the intracellular organelle, membrane-bound organelle, etc. (Figure 3B). The top-most upregulated genes are involved in protein binding, organic cycle compound binding, etc. as evident from their molecular functions (Figure 3C). Apart from these, the top-most upregulated genes are involved in the metabolic and disease pathways, like Alzheimer’s disease pathway, Huntington disease pathway, etc. (Figure 3D). In Biological Processes section for the top downregulated genes, it is shown that they are involved in the multicellular organismal process, developmental process, localization process, etc., (Figure 4A).

The downregulated genes are present in the cytoplasm, cell periphery, plasma membrane, etc., as shown in the cellular component (CC) section of the ontology terms. (Figure 4B) and the top-most downregulated gene are involved in protein binding, anion binding, enzyme binding as shown in their molecular functions (Figure 4C). Furthermore, KEGG pathway analysis shows the top downregulated genes are involved in cancer pathways, MAPK kinase signaling pathway, Ras signaling pathway, etc., (Figure 4D).

### 3.3. Protein–Protein Interaction Network Construction and Downstream Analysis

**Figure 5:**
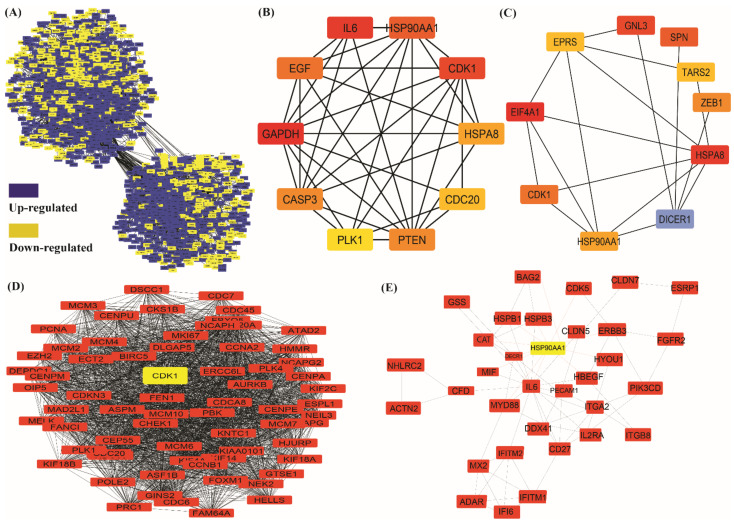
(**A**) Protein–protein network of DEGs. Blue color node showing upregulated genes and yellow color node showing downregulated genes of non-small cell lung cancer vs. normal. (**B**) Top-10 hub, and (**C**) bottleneck genes of PPI network retrieved from significant modules (dark color showing higher value in analysis, all are upregulated except the blue which represents downregulated genes) (**D**,**E**) Top-two modules after integrated analysis of PPI network (**D**) Modules 1 (**E**) Module 2. Node showing genes, lines denoted interaction between genes.

In our analysis, a total of 2700 DEGs were submitted to STRING for the construction and analysis of the network. The network analysis shows that the protein–protein interaction network has 2637 interacting nodes and 31,349 edges (Figure 5A). Further analysis of this network was executed using Cytoscape. After analyzing, we found the top-10 hub genes (genes with highest degrees), GAPDH, IL6, CDK1, HSP90AA1, EGF, PTEN, CASP3, HSPA8, CDC20, and PLK1 as per the decreasing order of degree values. Also, we found top 10 bottleneck genes EIF4A1, HSPA8, GNL3, SPN, CDK1, ZEB1, HSP90AA1, EPRS, TARS2, and DICER1. as per the decreasing order of highest bottleneck score (Figure 5B,C and Supplementary File S4).

A total of 65 modules were identified using the MCODE (Molecular Complex Detection) plug-in of the Cytoscape tool, out of which five cluster-significant modules (Modules with MCODE score ≥ 5 were considered as significant modules) were screened from the PPI network. Modules A had 68 nodes and 2078 edges with a 62.03 MCODE score, modules B had 161 nodes and 2417 edges with 30.213 MCODE scores, modules C had 31 nodes and 291 edges with 19.4 MCODE scores, modules D had 36 nodes and 308 edges with 17.6 MCODE score, modules E was having 19 nodes and 150 edges with 16.66 MCODE scores, etc. The list of MCODE scores and network parameters are given in Supplementary File S5 and the details of top twenty genes with highest degree, betweenness centrality, closeness centrality and eigen vector values are given in Supplementary File S6. (Figure 5D,E).

The topological properties of this network, probability of degree distribution P(k), neighborhood connectivity C_N_ (k), and clustering coefficient C(k), follow power-law characteristics as a function of k (Equation (1)). The negative value in γ of connectivity parameter shows disassortive nature of the network, and possibility of rich-club formation among the leading hubs. However, the roles of the leading hubs are still significant in regulating the NSCLC network.

(1) $$\left[\begin{array}{c} P\\ \;C\;\\ C_{N}\; \end{array}\right]=\left[\begin{array}{c} \kappa^{-\alpha }\\ \;\kappa^{-\beta }\;\\ \kappa^{-\gamma }\; \end{array}\right]\;;\;\left[\begin{array}{c} \alpha \;\\ \beta \;\\ \gamma \; \end{array}\right]\;\to \left[\begin{array}{c} 0.6257\;\\ 0.03\;\\ 0.227\; \end{array}\right]$$

(2) $$\left[\begin{array}{c} C_{B}\\ \;C_{C}\;\\ C_{E}\; \end{array}\right]=\left[\begin{array}{c} \kappa^{\varepsilon }\;\\ \kappa^{\eta }\;\\ \kappa^{\lambda }\; \end{array}\right];\;\left[\begin{array}{c} \varepsilon \\ \;\eta \;\\ \lambda \; \end{array}\right]\;\to \left[\begin{array}{c} 1.7\;\\ 0.104\;\\ 1.5\; \end{array}\right]$$

**Figure 6:**
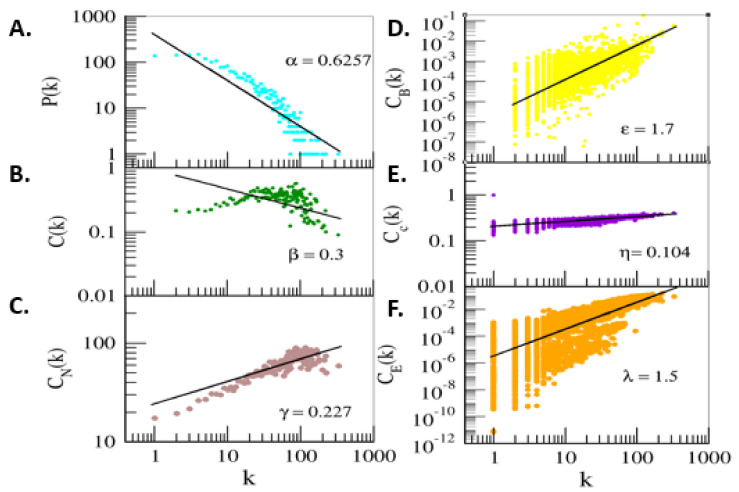
Topological properties of the network. (**A**) Probability of node degree distribution as function of degree k. (**B**) Clustering coefficient as function of degree k (**C**) Neighborhood connectivity as function of degree k. (**D**)Betweenness centrality as function of degree k. (**E**) Closeness centrality as function of degree k. (**F**) Eigenvector value as function of degree k.

Node degree distribution, clustering coefficient, and neighborhood connectivity were found to follow the hierarchical structure of the network. Centrality values, betweenness ($C_{B})$, closeness ($C_{C})$, and eigenvector ($C_{E})$ supported the network’s hierarchical nature. The positive value of exponents of these centrality parameters, shown in Equation (2), indicate the strong regulatory role of the leading hubs in the NSCLC network (Equation (2)). All six distributions of topological properties were fitted with power laws y = A_0_x^A1^ (Figure 6).

### 3.4. Key Regulator Gene Identification

**Figure 7:**
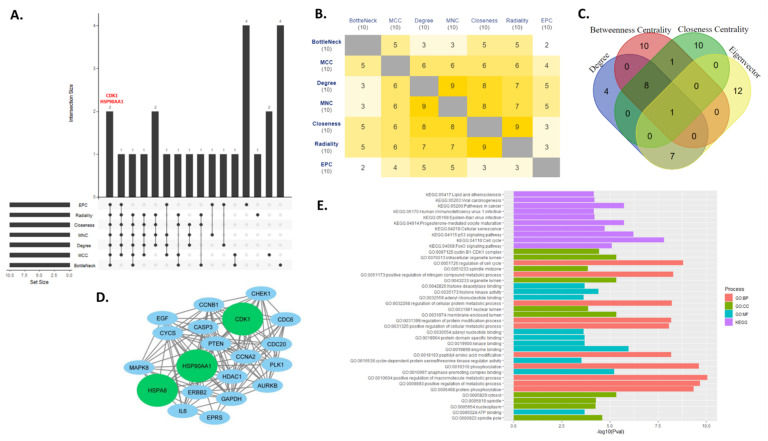
(**A**) Identifying common genes among seven topological properties. The first bar in the upset plot denotes two genes CDK1 and HSP90AA1 are common among top ten genes of each of the seven topological parameters. (**B**) Mutual intersections of each of the seven topological properties are shown as heat maps. (**C**) Intersections among top twenty genes having highest degree, betweenness centrality, closeness centrality and eigenvector values. (**D**) regulatory network of top 20 hub genes (top twenty genes ranked by degrees). (**E**) *p*-values are used to color a bar graph of enriched terms for a regulatory network of top-20 hub genes.

To identify key regulators in the PPI network, top ten genes from each of the six topological properties were taken to compare with each other. After analyzing the topological properties (closeness centrality, maximal clique centrality (MCC), Maximum Neighborhood Component (MNC), radiality, EPC (Edge Percolated Component and bottleneck), and tracing them to top ten hub genes (genes with top ten degrees) CDK1 (Cyclin-dependent kinase 1) and HSP90AA1 (heat shock protein 90 alpha family class A member 1) were found common for all topological properties. This suggests significant regulatory roles of CDK1 and HSP90AA1 in non-small cell lung cancer. Figure 7A shows the upset plot for all six topological properties and degree and genes common to them. The first bar shows two genes CDK1 and HSP90AA1 are common to all six topological properties and degree. Common genes between any two topological properties are shown as heatmap (Figure 7B). As an extension to this we also checked top twenty genes having highest degree, betweenness centrality, closeness centrality, and eigen vector. The common genes for each pair are shown in Figure 7C. 

Most of the hub genes in the network like GAPDH, IL6, CDK1, PTEN, etc. are either associated with non-small cell lung cancer itself or other forms of cancers. Therefore, we further checked the protein-protein interaction network and ontology terms of top twenty hub genes. We created a small network of these top 20 hub genes to check their interactions (Figure 7D). Further we analyzed the enrichment of the genes involved in the network. Top ten ontology terms for each of biological processes, cellular component, molecular function and KEGG pathways of these genes are given in Figure 7E and Supplementary File S6. We noted that p53 signaling pathway (KEGG:04115), FOXO signaling pathway (KEGG:04068), cyclin B1-CDK1 complex (GO:0097125), regulation of cell cycle (GO:0051726), histone deacetylase binding (GO:0042826) were some of the significant pathways for these top 20 hub genes.

### 3.5. Survival Plot Analysis of the Driver Gene

**Figure 8:**
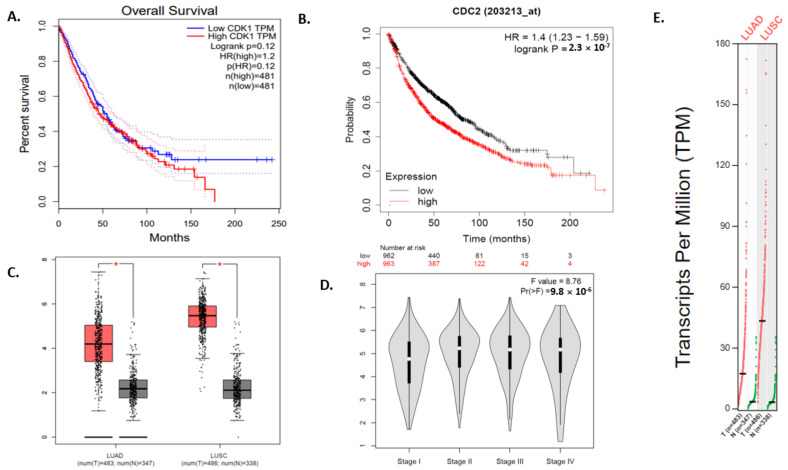
The survival, expressional and pathological analysis of CDK1. (**A**) Survival plot for CDK1 obtained from GEPIA (gepia.cancer-pku.cn). (**B**) Survival plot of CDK1 (CDC2) from KM-Plotter. (**C**) Expression profile of CDK1 in normal (grey) and tumor (red) samples of LUAD and LUSC datasets. (*p* < 0.05) (**D**) Pathological stage condition for CDK1 genes using LUAD and LUSC datasets. (**E**) Expression of CDK1 gene based on RNA-seq data from the TCGA database assessed by TPM (Transcripts per million) from GEPIA. One * (star) signifies *p* value less than 0.05. It is updated in the text.

**Figure 9:**
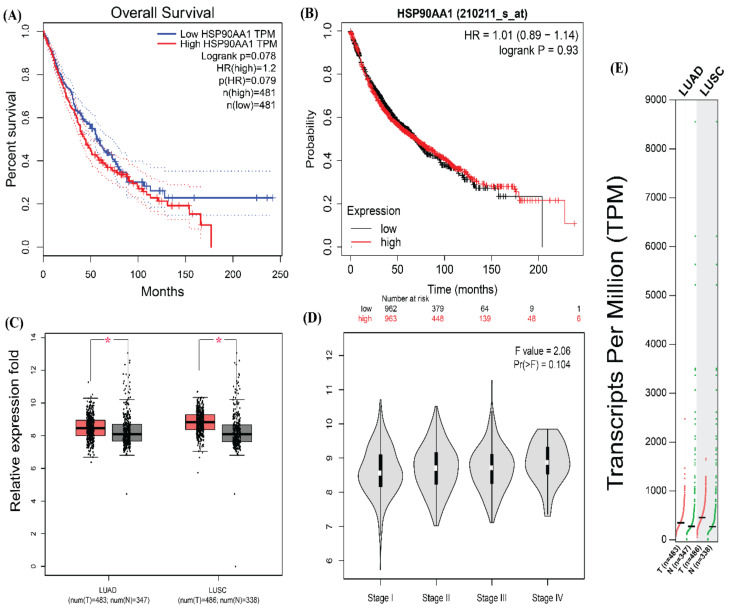
The survival, expressional and pathological analysis of HSP90AA1. (**A**) Survival plot for HSP90AA1 obtained from GEPIA (gepia.cancer-pku.cn). (**B**) Survival plot of HSP90AA1 from KM-Plotter. (**C**) Expression profile of HSP90AA1 in normal (grey) and tumor (red) samples of LUAD and LUSC datasets (*p* < 0.05) (**D**) Pathological stage condition for HSP90AA1 genes using LUAD and LUSC datasets. (**E**) Expression of HSP90AA1 gene based on RNA-seq data from the TCGA database assessed by TPM (Transcripts per million) from GEPIA. (Survival analysis is done in GEPIA using log-rank test (Mantel–Cox test), for the hypothesis evaluation.) One * (star) signifies *p* value less than 0.05. It is updated in the text. One * (star) signifies *p* value less than 0.05. It is updated in the text.

The mRNA expression of CDK1 and HSP90AA1 were found significantly connected with overall survival through the GEPIA online software (Figure 8A and Figure 9A). The survival plot of both CDK1 (CDC2) and HSP90AA1 genes were obtained from the GEPIA tool and were analyzed. Higher expression of both the genes CDK1 and HSP90AA1 was found to be related to poor overall survival. The Kaplan Meier (KM) plot also showed that CDK1 and HSP90AA1 expression were significantly associated with OS (Figure 8B and Figure 9B).

Moreover, the GEPIA tool was used to validate the expression of CDK1 and HSP90AA1 gene expression between lung cancer and control tissue in the LUAD and LUSC cohort from TGCA data. The mRNA expression of both genes CDK1 and HSP90AA1 were significantly upregulated in both the datasets, LUAD and LUSC, between lung cancer and non-cancer patients (Figure 8C and Figure 9C). Further, the relationship between the expression of CDK1, HSP90AA1, and tumor stage (pathological stage plot) in lung cancer patients was evaluated using GEPIA. The data revealed that CDK1 expression levels showed a strong association with the stage of tumor in lung cancer patients as well as HSP90AA1 also showed a strong association with the stage of tumor in lung cancer patients (Figure 8D and Figure 9D).

**Figure 10:**
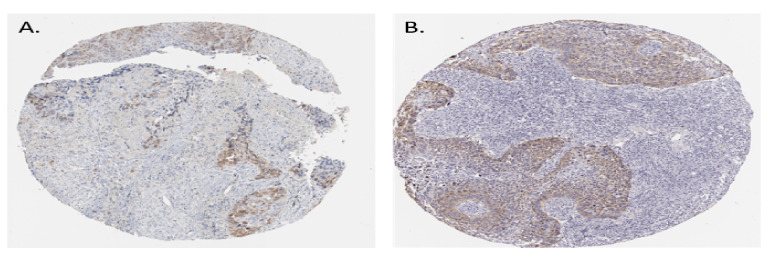
Histological images of CDK1 and HSP90AA1 in squamous cell carcinoma. (**A**) The sample is from Male, age 54 (HPA003387), Lung (T-28000) Squamous cell carcinoma, NOS (M-80703), Patient id: 1727 and for HSP90AA1 (**B**) The sample is from Male, age 68 (CAB002058), Lung (T-28000) Squamous cell carcinoma, NOS (M-80703), Patient id: 1048.

The TPM’s (transcripts per million) analysis of RNA-seq data from GEPIA with default parameters revealed that CDK1 and HSP90AA1 were overexpressed in both dataset LUAD and LUSC. (Figure 8E and Figure 9E). We then analyzed the protein expression patterns of these CDK1 and HSP90AA1 in NSCLC tissues by using the HPA database (Figure 10).

## 4. Discussion

Non-Small Cell Lung Cancer (NSCLC) is highly malignant with only 5-year survival rate [40,41] and significant percentage of cancer-related deaths [42]. Network analysis and functional enrichment are well known techniques to find novel significant regulatory genes for any biological network [43,44,45,46,47,48,49,50]. In our study we tried to identify potent biomarkers, by analyzing the DEGs between lung cancer and normal lung tissue samples of 550 Lung Cancer patients in a total of eight datasets from Gene Expression Omnibus (GEO). According to our functional enrichment analysis, the cell cycle, metabolic pathways, pathways in cancer, and the MAPK signaling pathway were found to be well associated to the pathogenesis of NSCLC. This study revealed two significant genes, CDK1 and HSP90AA1, as key regulators of NSCLC. It was found that the expression of both CDK1 and HSP90AA1 were upregulated in NSCLC, and the expression of both genes was validated in LUAD and LUSC cohort independently. Also, CDK1 and HSP90AA1 were linked to overall survival in a Kaplan–Meier study (OS).

CDKs (cyclin-dependent kinases) are crucial proteins that regulate the cell cycle [51]. However, the cell cycle is an essential and strictly controlled mechanism that controls cell proliferation and cell growth and any deviation from normal cell cycle regulation results in uncontrolled proliferation and cancer [52]. Malignancy is indicated by the absence of cell-cycle regulation [53]. Several therapeutic approaches have been developed to regulate the cell cycle in cancers [54]. CDK1 is the catalytic subunit of a protein kinase complex that triggers cell division. The transformation from the G1 to the S phase of the cell cycle and G2 to mitotic phase of the cell cycle is regulated by CDK1 [51]. CDK1 activity is dysregulated frequently in various cancers. The latest data reveals that CDK1 is a promising diagnostic and prognostic biomarker and a target for lung cancer since it participates in cell cycle progression and dysregulation [55]. A previous study suggested that targeting the CDK1 has reduced apoptotic resistance in colorectal cancer [56]. Another study showed that protein phosphatase1alpha mobilization of CDK1 mediated by a positive feedback loop had been shown to drive androgen receptors in prostate cancer [57]. The study also speculated that accumulated cytoplasmic Cdk1 is correlated with the growth and survival rate of epithelial ovarian cancer [58]. Kubo et al. observed that the prognosis of stage I and stage II non-small cell lung cancer are found to be dependent on cyclin-dependent kinases [59]. Another study showed that lung carcinogenesis is promoted by the activation of the GP130/STAT3 signaling pathway iron-dependent activity of CDK1 [60]. It was reported by Shi et al. that predictive and prognostic values of CDK1 and MAD2L1 have been described in lung adenocarcinoma as higher expression of CDK1 is correlated to poor survival and higher chances of cancer recurrences [61]. A Cdc2/Cdk1 inhibitor, purvalanol A, has been shown to enhance the cytotoxic effects of taxol by Op18/stathmin in in-vitro NSCLC [62].

Furthermore, Dinaciclib has been shown to downregulate lung cancer proliferation by inhibiting cyclin-dependent kinases 1 and 2. In contrast, Cucurbitacin-D-induced upregulation of CDK1 mRNA level has been shown to arrest the proliferation of non-small cell lung cancer [63,64]. Therefore, Li et al. and his co-worker concluded that CDK1 appears as a prognostic biomarker and therapeutic target in lung cancer [65]. Nevertheless, further characterization of CDK1 as a biomarker of NSCLC is essential for the better treatment of lung cancer. Our study uses a network theoretical approach to decipher the complex architecture of the NSCLC network. It identifies CDK1 as a potential biomarker and therapeutic target in the progression of non-small cell lung cancer.

Heat-associated protein 90, such as heat shock protein (HSP90), is a well-known and universal protein. HSP90 consists of a flexible homodimer, and its molecular structure consists of three regions: the N-terminal domain, the middle domain, and the C-terminal domain. The gene of HSP90AA1 (commonly known as HSP90) is situated on the chromosomes 14q32.2 [66]. In current cancer therapy, HSP90 is the center of attraction for its ability to inhibit multiple signaling pathways simultaneously [67]. A study reported by prominent scientists that IPI 504, a potent HSP90 inhibitor, has shown therapeutic response in patients with NSCLC, especially those with anaplastic lymphoma kinase gene rearrangements [68]. HSP90 has been highly expressed in multiple cancer like lung, ovarian, endometrial, and pancreatic cancer, additionally oropharyngeal squamous cell carcinoma (OSCC) and various myeloma [69,70,71,72]. The latest study revealed that high HSP90 expression was a poor prognosis marker in different cancers such as lung cancer, melanoma, esophageal cancer, bladder cancer, and leukemia [73,74,75,76]. Apart from these, HSP90 has been confirmed to play a role in a variety of cancers as well as overexpression of HSP90 has been suggested to improve carcinogenesis and influence patient prognosis [77,78,79,80,81]. The study found that the presence or absence of HSP90 expression affects the OS rate of NSCLC patients. Another study found that HSP90AA1 protein product HSP90α is thought to play a key role in tumor invasion and migration regulation [82,83]. Our study can conclude that high HSP90AA1 mRNA expression is associated with a poorer prognosis of OS in all NSCLC patients. Current research suggested that HSP90 could be a critical drug target for the treatment of NSCLC. For example, mitochondrial HSP90 network repression can be an effective treatment for refractory tumors [84] as well as the introduction of HSP90 inhibitor 17AAG prevents epidermal hyperplasia and OSCC [85]. HSP90AA1 affects the patient’s reaction with systemic lupus erythematosus to glucocorticoids and the lipopolysaccharide-induced inflammatory response, including tumor necrosis factor secretion by monocytes, according to previously published findings [86]. Moreover, the role of HSP90AA1 in cancer pathogenesis is a hot topic of research right now. According to a previous study, high HSP90AA1 expression was an independent factor correlated with mortality in breast cancer patients (triple-negative and human epidermal growth factor receptor 2-negative/estrogen receptor-positive subtypes) [87].

## 5. Conclusions

In conclusion, our study identified multiple important genes, including CDK1 and HSP90AA1 which showed differential expression in non-small cell lung cancer patients compared to control through DEG analysis and network building. Our integrated analysis suggested that CDK1 and HSP90AA1 could be significant potential biomarkers for the diagnosis and prognosis of NSCLC. Both CDK1 and HSP90AA1 could be potential therapeutic drug targets in lung cancer treatment. However, some drawbacks to the current research are needed to be addressed. Moreover, the current findings were extracted by applying bioinformatics techniques, therefore efficient experimental research is required to fully understand the underlying molecular mechanisms in future.

## Figures and Tables

**Table 1:** List of datasets used in the meta-analysis.

Microarray Datasets	Platforms	Control	Cases	References
GSE19188	GPL570	65	91	[23]
GSE118370	GPL570	6	6	[24]
GSE10072	GPL96	49	58	[25]
GSE101929	GPL570	34	32	[26]
GSE7670	GPL96	27	27	[27,28]
GSE33532	GPL570	20	80	[29]
GSE31547	GPL96	20	30	-
GSE31210	GPL570	20	226	[30,31]

## Data Availability

The GSE files are publicly available. The codes can be shared upon reasonable requests to the corresponding author Md. Zubbair Malik.

## Supplementary Materials

The following supporting information can be downloaded at: https://www.mdpi.com/article/10.3390/jpm12030393/s1, Supplementary File S1: Meta-analyzed data of all datasets; Supplementary File S2: Gene enrichment analysis of upregulated DEGs in lung datasets; Supplementary File S3: Gene enrichment analysis of down regulated DEGs in lung datasets; Supplementary File S4: Top-ten topological properties of genes; Supplementary File S5: The details of modules identified by MCODE; Supplementary File S6: List of top twenty genes according to rank of topological properties.
